# “Age Related Differences in the Biology of Chronic Graft-Versus-Host Disease After Hematopoietic Stem Cell Transplantation”

**DOI:** 10.3389/fimmu.2020.571884

**Published:** 2020-10-16

**Authors:** Geoff D. E. Cuvelier, Amanda Li, Sibyl Drissler, Amina Kariminia, Sayeh Abdossamadi, Jacob Rozmus, Jean-Pierre Chanoine, Bernard Ng, Sara Mostafavi, Ryan R. Brinkman, Kirk R. Schultz

**Affiliations:** ^1^ Pediatric Blood and Marrow Transplant Program, CancerCare Manitoba, University of Manitoba, Winnipeg, MB, Canada; ^2^ Michael Cuccione Childhood Cancer Research Program, British Columbia Children’s Hospital, University of British Columbia, Vancouver, BC, Canada; ^3^ Terry Fox Laboratory, BC Cancer, Department of Medical Genetics UBC, Vancouver, BC, Canada; ^4^ Department of Cell and Developmental Biology, University of British Columbia, Vancouver, BC, Canada; ^5^ Department of Pediatrics, Endocrinology and Diabetes Unit, British Columbia Children’s Hospital, Vancouver, BC, Canada; ^6^ Department of Statistics, Centre for Molecular Medicine and Therapeutics, British Columbia Children’s Hospital, University of British Columbia, Vancouver, BC, Canada

**Keywords:** chronic graft-versus-host disease, children, adolescent, adults, puberty, immune profile

## Abstract

It is established that pediatric hematopoietic stem cell transplant (HSCT) recipients have a lower rate of chronic graft-versus-host disease (cGvHD) compared to adults. Our group has previously published immune profiles changes associated with cGvHD of clinically well-defined adult and pediatric HSCT cohorts. Since all analyses were performed by the same research group and analyzed using identical methodology, we first compared our previous immune profile analyses between adults and children. We then performed additional analyses comparing the T cell populations across age groups, and a sub-analysis of the impact of the estimated pubertal status at time of HSCT in our pediatric cohort. In all analyses, we corrected for clinical covariates including total body irradiation and time of onset of cGvHD. Three consistent findings were seen in both children and adults, including elevations of ST2 and naive helper T (Th) cells and depression of NK_reg_ cells. However, significant differences exist between children and adults in certain cytokines, B cell, and T_reg_ populations. In children, we saw a broad suppression of newly formed B (NF-B) cells, whereas adults exhibited an increase in T1-CD21^lo^ B cells and a decrease in T1-CD24^hi^CD38^hi^ B cells. Prepubertal children had elevations of aminopeptidase N (sCD13) and ICAM-1. T_reg_ abnormalities in children appeared to be primarily in memory T_reg_ cells, whereas in adults the abnormalities were in naïve T_reg_ cells. In adults, the loss of PD1 expression in naïve T_reg_ and naïve Th cells was associated with cGvHD. We discuss the possible mechanisms for these age-related differences, and how they might theoretically impact on different therapeutic approaches to cGvHD between children and adults.

## Background

Pediatric hematopoietic stem cell transplantation (HSCT) recipients have a lower rate and possibly different presentations of chronic graft-versus-host disease (cGvHD) compared to adults (1). It has long been hypothesized that greater thymic function in children is the primary reason for the lower rate cGvHD, yet little human evidence after HSCT supports this hypothesis. Previous evaluations of cellular and plasma markers of cGvHD in adults, by our group and others, have identified 3 primary cellular populations that characterize cGvHD, including CD21^lo^ B cells, NK_reg_ cells, and naïve T cells; as well as consistent changes in ST2 (2–6). Our group recently concluded the Applied Biomarkers of Late Effects of Childhood Cancer (ABLE)/Pediatric Blood and Marrow Transplant Consortium (PBMTC) 1,202 study, which included 302 pediatric transplant patients, 52 of whom had cGvHD (7, 8). This has allowed us to compare and contrast potential differences between children and adults in the immune cell and plasma cytokine profiles seen in our previous adults studies that correlate with the development of cGvHD.

Puberty is the result of activation of the hypothalamo-pituitary-gonadal axis and, as a consequence, of the increased production of androgens and estrogens usually starting between the age of 8 and 13 years in girls and 9 and 14 years in boys (9). Onset of puberty is associated with a decline in thymic function, and possibly splenic function as well (10, 11). Yet, the exact differences that age and puberty have on the development of immune reconstitution post-HSCT, GvHD, and the induction of immune tolerance are still incompletely studied. Comprehensive approaches to immune profiling, evaluation of thymic and splenic function, as well as the impact of sex hormones on the development of cGvHD are needed.

In this manuscript, we use our ability to perform broad immune cell (including T-, B-, and NK-cell populations) and plasma cytokine profiling to examine for differences in cGvHD markers between prepubertal children, pubertal adolescents, and adults. Based on these preliminary analyses, we develop hypotheses that could explain how cGvHD might be influenced by the recipient’s age and pubertal status at the time of HSCT and provide insight into how cGvHD may be biologically different between children and adults.

## Methods

### Study Populations

#### Adult Biomarker Studies

Samples from patients ≥18 years of age were collected as part of a Canadian Institutes of Health Research funded biomarker companion study based at the BCCH Research Institute, including 9 Canadian adult HSCT centers [all members of the Canadian Blood and Marrow Transplant Group (CBMTG)], 2 US centers, and 1 Saudi Arabian HSCT center. The companion study included patients enrolled on the CBMTG 0601 and 0801 trials as previously described (4, 12). Peripheral blood samples were collected on day 100 ± 14 days after transplantation, as well as at the time of cGvHD diagnosis, and evaluated for cellular and plasma markers. All samples were obtained after informed consent with institutional research ethics board approval.

#### Pediatric ABLE/PBMTC 1202 cGvHD Biomarker Study Design

Twenty-seven pediatric transplant centers (6 Canadian, 20 US, and 1 Austria) enrolled 302 patients <18 years of age between August 2013 to February 2017. All sites had institutional ethics board approval. The clinical study was described in detail (7). Centers assessed children for cGvHD according to the 2005 National Institutes of Health Consensus Criteria, with review by a central study adjudication committee composed of experts in cGvHD. Samples were evaluated by a broad immunophenotyping and plasma cytokine strategy (8).

#### Assessment of Pubertal Status in Children

Since our pediatric study did not include formal Tanner staging when designed, we evaluated the impact of the estimated pubertal status at the time of HSCT on the later development of cGvHD by approximating pubertal development, based upon the average signs of visible pubertal changes that reflect the secretion of gonadal hormones, corresponding to breast Tanner stage 2 in girls and genitalia (penis) Tanner stage 3 in boys (13). Based on these assumptions, we estimated a “puberty cut-off” (pre-pubertal versus pubertal) in girls as reflecting the 50^th^ percentile for Breast Tanner stage 2, occurring at 10.9 years (95% range; 8.9 to 12.9 years), and for boys as the 50^th^ percentile in penis Tanner stage 3 occurring at 12.4 years (95% range; 10.1 to 14.6 years) (13).

### Shipping of Samples

Two different types of tubes were used for sample collection: heparinized tubes for plasma (BD Vacutainer) and Cyto-chex^®^ BCT tubes, STRECK. INC. (Canada distributor: Inter Medico, Markham, ON, Canada) tubes for immunophenotyping. All peripheral blood samples were shipped to the Transplantation Applied Biomarkers laboratory at BC Children’s Hospital Research Institute in Vancouver, BC, Canada *via* FedEx overnight priority shipping (delivered within 24 h after blood collection). Plasma isolation and storage: upon sample delivery, plasma was isolated from blood cellular component by primary centrifugation. Plasma aliquots were kept frozen at -80°C until usage. The tubes were shipped at room temperature overnight and phenotyping performed on the same day of sample delivery.

### Phenotyping Procedure

Five panels were designed to look for different sub-populations in T, B, dendritic, and NK cells. All antibodies, corresponding conjugated dyes, clones, and vendors as previously described [(8), **Supplemental Table 3**]. One hundred microliter of blood was used for all panels except for the T_reg_ panel where 200 ul of blood was used. Samples were stained in the dark for 12 min at room temperature (RT) followed by treatment with fix/RBC lyze solution (eBiosceinces, Thermo Fisher Scientific, Waltham, US). For intracellular staining, cells were made permeable using BD Perm II solution (BD Biosciences Mississauga, Canada). Flow cytometry data were acquired using BD LSR Fortessa X-20 Special Order four channel flow cytometer (BD Biosciences, San Jose, CA, US). A minimum of 300,000 events were acquired for all panels. Instrument settings was also standardized using SPHERO™ Rainbow Calibration particles 6 peaks (Sphereotech, Lake Forest, IL, US) to adjust laser power drifts over time. FCS files were analyzed using Kaluza software v2 (Beckman Coulter, INC. Mississauga, Canada). Flow cytometry accuracy, reproducibility was ensured by the detailed approaches as previously described (8).

### Cytokine Measurement

Samples were collected and shipped as previously described (4, 8). Platelet depleted plasmas were isolated and frozen within 24 h of collection, as previously described (4). Batches of plasma samples were thawed and eleven cGvHD-associated markers were analyzed in both the adult and pediatric cohorts, including ST2, Osteopontin, sBAFF, sCD25, TIM-3, MMP3, ICAM-1, CXCL10, CXCL9, CXCL11, and soluble aminopeptidase N (sCD13). Reg3alpha was measured in the pediatric population only. CXCL9 and CXCL11 were measured using electrochemiluminescence dual-plex plate (Meso Scale Diagnostics LLC, Gaithersburg, US). sCD13 was measured using colorimetric assay based on enzymatic activity, as previously described (4). The remaining cytokines were measured by standard colorimetric ELISA (RnD Systems, Minneapolis, US). We found a high accuracy, reproducibility, and linearity for all assays measuring soluble biomarkers and a high stability of analytes upon 24 shipment as have been previously described in adults (4) and children (8).

### Statistical Analysis of Results

Flow cytometry data was pre-processed by removing margin events, compensating the data, applying a logicle transform and using flowCut (14) to eliminate artifacts caused by poor flow. Files were then gated based on a designated gating strategy using flowDensity (15). After preprocessing the flow cytometry data, the flowType pipeline was used to identify cell populations as previously described (3). We looked at the 2-grouping cGvHD- versus cGvHD+. We conducted a statistical analysis of the cell frequencies as a percentage of their respective parent populations for all populations in pre-determined gating strategy. All three criteria were required to highlight biologically relevant markers including: a) p ≤0.05, b) receiver operator curve (ROC) area under the curve (AUC) ≥0.60, and c) effect ratio of ≥1.3 or ≤0.75. The p-value of each marker was estimated based on the Wald test. ROC AUC was computed by estimating the true positive rate (proportion of cGvHD or late aGvHD correctly classified) against the false positive rate (proportion of controls falsely classified as cGvHD or late aGvHD) for different marker thresholds. The effect ratio was calculated as the average marker value of patients with cGvHD (or late aGvHD) divided by the average marker value of controls. For the T cell analysis, when using the flowType pipeline for the 2-grouping, we found X/Y-values <0.05. Of the immunophenotypes with significant p-values, we selected those with ROC AUC ≥0.6 and effect ratios of ≥1.3 or ≤0.75 for analysis with RchyOptimyx (16) in order to find the minimal and optimal set of immunophenotyping markers for the diagnosis of cGvHD.

Patients were assigned as having cGvHD or as a control in the analysis, as we previously have described (8). As our analyses were exploratory, no statistical adjustments were made for multiple testing. Given that there were numerous tests conducted, the probability of a Type I error likely exceeded 0.05, but this was moderated by the additional ROC AUC and effect ratio criteria. Because our data base did not include Tanner staging, we examined the impact of the puberty status at the time of HSCT on the later development of cGvHD by estimating the onset of puberty based where the average age of onset associated with an increase in the production of gonadal hormones that is sufficient to cause an increase in growth in height velocity: with breast Tanner stage 2 in girls and genitalia (penis) Tanner stage 3 in boys (13). Based on the puberty “cut-off” established above (13), each of the pediatric ABLE cohort (cGvHD, aGvHD, and controls) were divided <10.9 and 12.4 years and ≥10.9 and 12.4 years for boys and girls, respectively, and analyzed for each age group. The following clinical variables were modeled as confounding factors in the logistic regression model: a) prophylaxis or treatment with either alemtuzumab or ATG, b) prophylaxis or treatment with rituximab, c) recipient age, d) the use of a peripheral blood donor product or not, e) whether the donor was HLA-identical or not. In addition, all analyses were corrected for whether patients received TBI and the time of onset of cGvHD. All analyses were performed using MATLAB (MathWorks, Natwick, Mass, USA) and R (17).

## Results

### Description of the Pediatric and Adult Population Utilized in the Comparison of Immune Profiling in cGvHD

For an initial comparison of immune profile differences between the adult and pediatric cohorts, we utilized the published results of 2 previously evaluated cohorts. The adult cohort (**Table 1**; N = 107) has been previously evaluated for B cell population profiles (18) and NK, T cell, and cytokine, populations at the onset of cGvHD (4). The pediatric cohort was from the later ABLE studies (**Table 1**; N = 302), and to date, only the analyses of the day 100 samples have been completed and described (8). The two cohorts are representative of the type of transplants performed in adults and children. While all HSCTs in the adult cohort were for malignant conditions, only 59% were in the pediatric cohort (**Table 1**). The pediatric cohort was characterized by greater use of cord blood as a donor source, compared to G-CSF mobilized peripheral blood progenitor cells (PBPC) being used more frequently in the adult cohort. Sibling donor transplants were more common in the pediatric cohort. The tissue distribution of cGvHD in the two cohorts was different, in that skin and lung cGvHD was more common in the adult population compared to the pediatric cohort. Atypical cGvHD presentations not associated with the diagnostic criteria for National Institutes of Health cGvHD were relatively frequent (21.7%) in the pediatric cohort, but this data was not reliably collected in the adult cohort to allow a comparison.

**Table 1:** Baseline Characteristics of the pediatric (ABLE) and adult cohorts evaluated in these analyses^3^.

Characteristic	Pediatric Cohort^1^(Overall Percentages of Entire Evaluable Cohort n = 243)		Adult Cohort^2^(Overall Percentages of Entire Evaluable Cohort n = 107)	
	No Late Acute GVHD or Chronic GVHD (n = 132)	Chronic GVHD (n = 51)	No cGvHD (n = 63)	cGvHD(n = 44)
**Diagnoses**				
**Malignant**	78 (59)	41 (80)	63 (100)	44 (100)
ALL	33 (25)	18 (36)	9 (14)	7 (16)
MDS/AML	37 (28)	15 (30)	25 (40) 9 (14)	11 (25) 12 (27)
Mixed Lineage Acute Leukemia/Other	0 (0)	1 (2)	2 (3)	1 (2)
NHL	4 (3)	3 (6)	10 (16)	7 (16)
JMML	3 (2)	1 (2)	0 (0)	0 (0)
CML	1 (1)	3 (6)	4 (6)	1 (2)
CLL	0 (0)	0 (0)	2 (3)	2 (5)
MM	0 (0)	0 (0)	2 (3)	3 (7)
**Non-Malignant**	54 (41)	10 (20)	0 (0)	0 (0)
**Sex**				
Male (55.6%)	72 (55)	33 (65)	34 (54)	26 (59)
Female (44.4%)	60 (46)	18 (35)	29 (46)	18 (41)
**Age at Transplant**				
Median Age (Years, Range)	9.3 (0.2–17.9)	11.9 (2-18)		
<50 years			25 (40)	16 (36)
≥50 years			38 (60)	28 (64)
**Donor and HLA Match**				
HLA-Matched Family Donor (8/8)	54 (41)	8 (16)	17 (27)	19 (43)
Haploidentical Family Donor (with PTCy)	2 (2)	1 (2)	0 (0)	0 (0)
HLA-Matched Unrelated Donor (8/8)	48 (36)	22 (43)	17 (27)	14 (32)
HLA-Mismatched Unrelated Donor (≤7/8)	9 (7)	12 (24)	29 (46)	11 (25)
Cord Blood Matched and mismatched	19 (12)	8 (16)	0 (0)	0 (0)
**Stem Cell Source**				
Bone Marrow	94 (71)	25 (49)	4 (6)	3 (7)
PBSC	19 (14)	18 (35)	32 (51)	37 (84)
Cord Blood	18 (14)	7 (14)	27 (43)	4 (9)
Double Cord Blood	1 (1)	2 (3)	0 (0)	1 (2)
**Conditioning Regimen**				
**Myeloablative**	111 (84)	45 (88)	27 (43)	18 (41)
TBI 1200-1320 cGY +/- Other	33 (25)	19 (38)	19 (30)	10 (23)
Chemotherapy + 200-400 cGY TBI	7 (5)	1 (2)	0 (0)	0 (0)
Myeloablative w/o TBI	71 (5)	25 (50)	8 (13)	8 (18)
**Reduced Intensity or Non-myeloablative**	21 (16)	6 (12)	36 (57)	26 (59)
**GVHD Prophylaxis**				
CNI + MTX/MMF ± Sirolimus	115 (88)	50 (97)	48 (76)	38 (86)
CNI ± Sirolimus	0 (0)	0(0)	10 (16)	6 (14)
PTCy + CNI + MMF	5 (4)	1 (2)	0 (0)	0 (0)
CNI + Steroid	1 (1)	0 (0)	0 (0)	0 (0)
Other	11 (8)	0 (0)	5 (8)	0 (0)
**History of Acute GVHD**				
None	87 (66)	8 (16)	27 (43)	22 (50)
Yes	45 (34)	43 (84)	36 (57)	22 (50)
**cGvHD Organ involvement in those affected by cGvHD**				
Skin involvement		43.1%		61%
Oral involvement		62.7%		66%
GI involvement		39.2%		23%
Eye involvement		29.4%		45%
Joint involvement		5.9%		15%
Lung involvement		23.5%		47%
Liver involvement		27.5%		51%
Genital involvement		2%		16%
Other Pericardial effusion Eosinophilia ITP Nephrotic syndrome Cardiomyopathy Neuropathy		21.7%		This data was not collected

^1^ Summarized data from the ABLE pediatric cohort (N = 183) as published (7, 8) ^2^ Summarized data from the adult cohort (N = 107) as published (3, 19); ^3^These two cohorts were used for both the summary of the known published results presented in **Table 2** as well as the prospective analyses presented in **Figures 1** **–** **3**.

### B Cell, NK Cell, and Cytokine Differences Between Adult and Pediatric Populations in our Previously Published Studies

We have previously analyzed these two cohorts separately and published the results (4, 8, 18). While the pediatric analysis is only focused on immune profiles at day 100 post HSTC (8) and measured before the onset of cGvHD, and published adult results (4) were measured at the onset of cGvHD, we felt we could identify age-related patterns to guide additional comparisons of the two cohorts. We have previously performed comprehensive analysis of the B cell profiles in children (8) and adults (18) in the same laboratory and utilizing similar immune profiling strategies. For a cell population to be considered increased or decreased, it had to meet our definition of being a biologically relevant marker meeting all three of the following criteria including a) p value ≥0.05, b) ROC AUC ≥0.60; and c) effect ratio of ≥1.3 or ≤0.75. If it did not meet all three of these rigorous inclusion criteria we excluded it from the comparison. Comparison of B cell immune profiles identified population similarities and differences between the adult and pediatric cohorts (**Table 2**). We found that both adults and children had decreases in newly formed B cell populations (NF-B cells). Only the pediatric cohort, however, had decreases in the T2 and T3 transitional B cell populations. By contrast, CD10^hi^CD38^hi^CD19^+^ B cells, another transitional B cell population commonly associated with the B_reg_ population, was depressed in adults at the time of cGvHD diagnosis, but not in children. Two B cell populations had opposite results between the pediatric and adult cohorts. CD21^low^ B cells were increased in adult cGvHD, but in children with cGvHD, were significantly decreased (**Table 2**). By comparison, an increase in unswitched memory/Marginal zone-like B cells in pediatric cGvHD was observed versus a significant decrease in switched memory B cells in adult cGvHD (**Table 2**). Both the adult and pediatric cohorts exhibited decreases in mature naïve B cells.

**Table 2:** Summary of Published Immune Profiling Studies Published by the BCCH group for B cells, NK cell and plasma marker populations associated with cGvHD in Separate Adult and Pediatric Cohorts.

Cell population		Pedia​tric (0–18 years; N = 241)^2^Day 100 in cGvHD	​Adult (≥ 18 years;N = 107)^1^Onset of cGvHD
**B cell populations**			
*T1 - Immature/Transitional B cell population consistent with Breg cells*	CD24^hi^CD38^hi^CD19^+^	NS	Decreased^4^
*CD21 low B cells*	CD21^lo^/CD19^+^	Decreased	Increased
*T2 transitional*	CD38^int^CD10^int^ of CD19^+^	Decreased	NS
*T3 transitional*	CD38^dim^CD10^lo^ of CD19^+^	Decreased	NS
*Mature Naïve*	CD27^-^ IgD^+^CD19^+^	Decreased	Decreased
*Unswitched memory/* *Marginal-zone like*	%CD27^+^IgD^+^ of CD19^+^	Increased	Decreased
*Classical switched memory*	%CD27^+^IgD^-^ of CD19^+^	NS	Increased
**NK cell populations**			
Regulatory CD56^bright^ NK cells		Decreased Before onset – day 100 Decreased at onset	NA Decreased at Onset^1^ Decreased in Donor cell infusion^3^
**Plasma markers**			
		Day 100	Onset of cGvHD
*Aminopeptidase N (sCD13)*		Increased	Increased but variable
*ST2*		Increased	Increased
*CXCL10*		NS	Increased
*CXCL9*		NS	Increased but variable
*ICAM-1*		Increased but variable	Increased but variable

^1^Rozmus (19) for adult data – N = 104**;** 44 with cGvHD onset (median of 207 days post-HCT; range 83–424 days) and 63 patients without cGvHD with sample collection a median of 194 days post-HCT (range 153–430 days**);** ^2^ Schultz KR (8) for pediatric data - N = 241 patients evaluated at day 100. ^3^Adult data on donor product infused as part of the CBMTG0601 trial (N = 79) (3). ^4^ To be defined as increased or decreased, the marker had to meet our definition of being a biologically relevant marker which included all of the following 3 criteria a) p value ≤0.05; b) ROC AUC ≥0.60; and effect ratio of ≥1.3 or ≤0.75.

In adults, we previously observed a similar decrease in NK_reg_ cells, both in the adult cGvHD cohort described in this paper (4) and in a separate adult donor cohort that had an evaluation of the infused donor product (3) (**Table 2**). In our pediatric cohort, we also saw a significant decrease in CD56^bright^ noncytolytic NK cells (8), NK cells that are consistent with regulatory NK cells (NK_reg_) (19, 20). Thus, unlike B cells, no age-related differences in the NK_reg_ population were observed, with decreases in NK_reg_ cells seen in both the adult and pediatric cGvHD cohorts.

Our group and others have observed plasma differences in a number of soluble factors between children and adults (4, 5). Our comparison of the ABLE pediatric cohort (8) found that ST2, in a similar manner to adults (5), is elevated in children (**Table 2**). Other cytokines such as CXCL10 and CXCL9 that were significantly elevated in adults with cGvHD (4, 6) could not be confirmed in the pediatric cohort. By comparison, aminopeptidase N (soluble CD13), originally found at cGvHD diagnosis by proteomic analysis in children (21), as well as ICAM-1, both appear increased in children with cGvHD.

### A Direct Comparison of T Cell Differences Between Adult and Pediatric Populations

**Figure 1:**
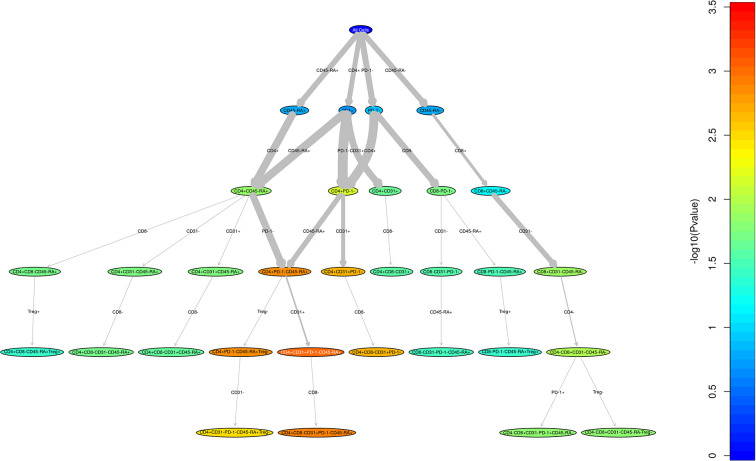
Unstructured analysis of adult and pediatric T cell populations in cGvHD. This RChyOptimyx plot depicts the results of the unstructured statistical analysis conducted to find combinations of markers which best predict cGvHD. Colors correspond to the p-values and the width of the arrows corresponds to the change in p-value after including an additional marker.

**Figure 2:**
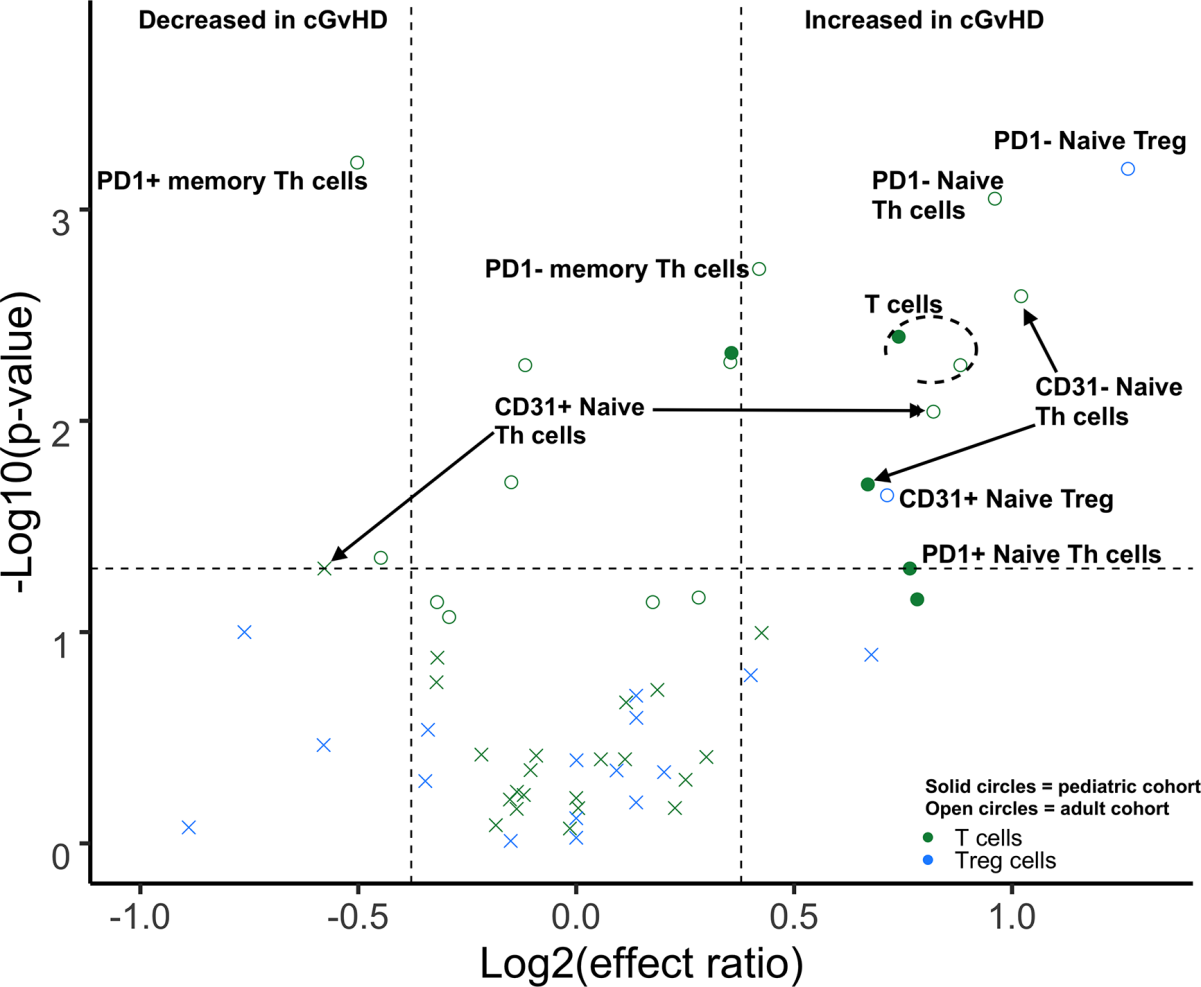
Differences of Day 100 adult and pediatric T cell populations in cGvHD. Volcano plots that met our definition of a biologically relevant markers for cGvHD were required to meet all 3 criteria of a i) p-value ≤0.05 (y-axis), ii) receiver operator curve (ROC) area under the curve (AUC) of ≥0.60 (circle: ≥0.60 and cross: <0.6), and iii) effect ratio ≥1.3 or ≤0.75 (x-axis). A circle that is on either the upper right quadrant (higher in cGvHD) or upper left quadrant (lower in cGvHD) was considered a significant markers whereas a cross in these same quadrants, while meeting the criteria for effect ratio and p value, did not have an ROC AUC ≥0.60. Cell populations are identified by color with green = T cells and light blue = T_reg_ cells. Solid circles = the pediatric cohort and open circles = the adult cohort.

We performed a direct comparison of the T cell populations evaluated at day 100 in both the adult and pediatric cohorts. Data was merged into a single data set with flow-cytometry reanalyzed to ensure identical gating between the two studies. An unsupervised comparison analyses (**Figure 1**) was then performed on the T cell populations at Day 100 after HSCT in the adult and pediatric ABLE cohort. Overall, T cell patterns appear to be more complex in the adult cGvHD patients compared to children at day 100 who would later go on to develop cGvHD (**Figure 2**; **Supplemental Table 1**). The only markers commonly affected in the same way between adults and children included an increase in overall CD3^+^ T cells and Naïve Th cells (CD31^-^CD3^+^CD4^+^CD45RA^+^). CD31^+^ (recent thymic emigrant or RTE) Naïve Th cells were also affected in both the pediatric and adult cohorts, but in opposite directions, being increased in adults compared to decreased in children (**Figure 2**). Additional changes in adults included an increase in the PD1^-^ Naïve Th cell population. While we could demonstrate no differences in T_reg_ cells in the overall pediatric cohort, we did see an increase in both PD1^-^ and CD31^+^ T_reg_ populations in the adult cohort. Memory Th cells were also significantly different in adults, with an increase in PD1^-^ memory Th cells and a decreased in PD1^+^ memory Th cells (**Figure 2**).

### Impact of Puberty Status Pre HSCT on Immune Profiles in cGvHD in the Pediatric Population

**Figure 3:**
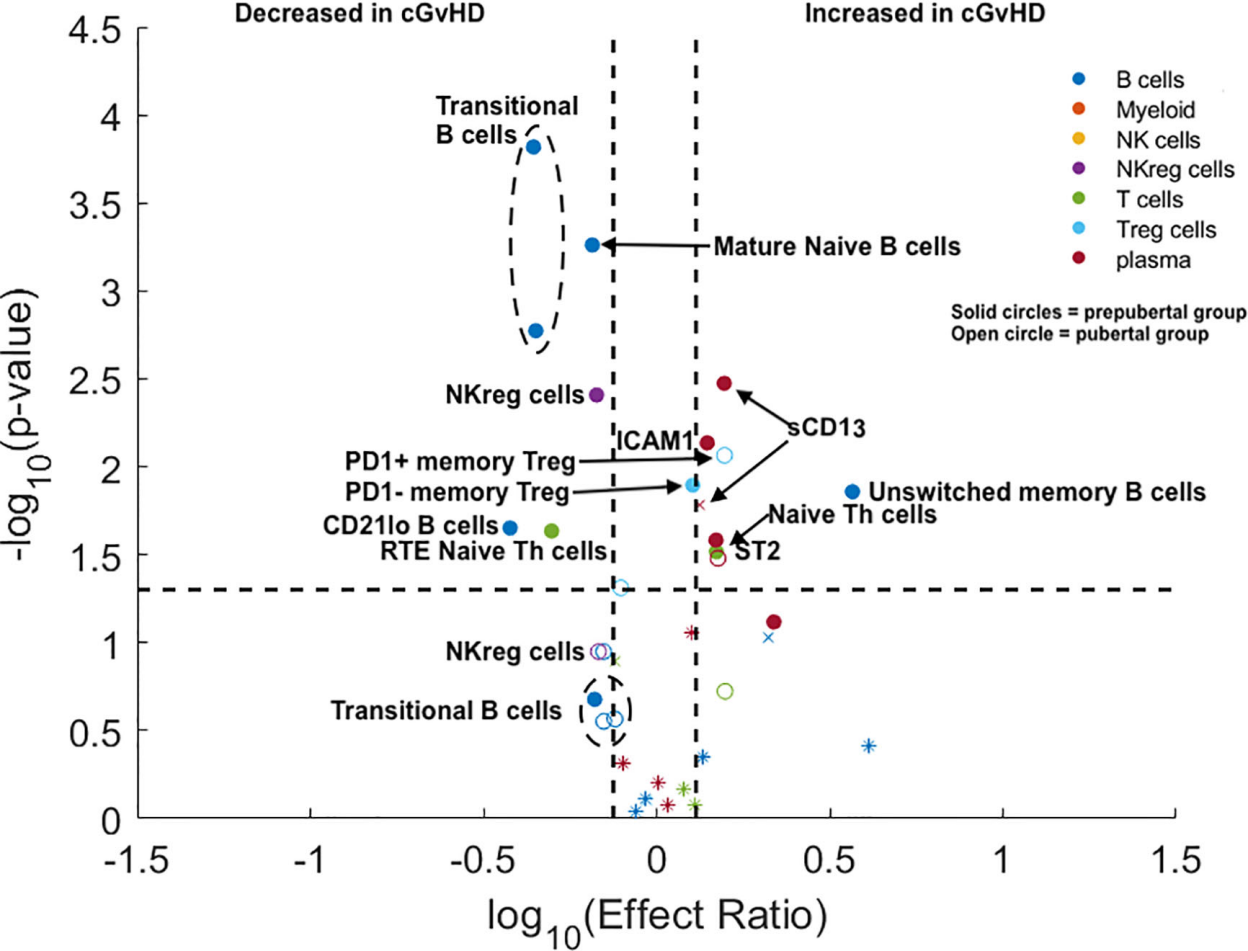
Evaluation of the impact of estimated pubertal status pre HSCT on cGvHD Immune Profile. Volcano plots that met our definition of a biologically relevant marker for cGvHD were required to meet all 3 criteria of a i) p-value ≤0.05 (y-axis), ii) receiver operator curve (ROC) area under the curve (AUC) of ≥0.60 (circle: ≥0.60 and cross: <0.6), and iii) effect ratio ≥1.3 or ≤0.75 (x-axis). A circle that is on either the upper right quadrant (higher in cGvHD) or upper left quadrant (lower in cGvHD) was considered a significant markers whereas a cross in these same quadrants, while meeting the criteria for effect ratio and p value, did not have an ROC AUC ≥0.60. Cell populations are identified by color with dark blue = B cells, orange = myeloid populations, yellow = NK cells, purple = NK_reg_ cells, green = T cells, light blue = T_reg_ cells, and dark red = plasma cytokines. Solid circles = the prepubertal group and open circles = the pubertal group. We note the following clinical variables were modeled as confounding factors in the logistic regression model: **(A)** prophylaxis or treatment with either alemtuzumab or ATG, **(B)** prophylaxis or treatment with rituximab, **(C)** recipient age, **(D)** the use of a peripheral blood donor product or not, **(E)** whether the donor was HLA-identical or not. The onset of puberty was estimated as 10.9 years in boys and 12.4 years in girls. The results are corrected for both the time of onset after HSCT and for the use of TBI.

The pediatric ABLE cohort included patients 0–18 years of age. We hypothesized that the differences we had identified between adults and children potentially would begin to change at the onset of puberty, with adolescent cGvHD markers becoming more adult-like after puberty. Based on standardized data for the onset of puberty in North American children (9), we estimated the average age of onset of Tanner stage 2 breast development in girls (10.9 years) and Tanner stage 3 penis development in boys (12.4 years) as reflecting secretion of gonadal hormones the point between being prepubertal and pubertal. We divided the <18 year old pediatric cohort from the ABLE study into a prepubertal (< 10.9 years for girls and < 12.4 years for boys) and pubertal age group based upon these age cut-offs, (≥ 10.9 years in girls and ≥ 12.4 years in boys) grouping and evaluated the impact of puberty at the time of HSCT on cGvHD immune profiles. All analyses were adjusted for a number of clinical factors including the impact of TBI and the time of onset of cGvHD (**Figure 3**). We evaluated the identical T cell populations identified in **Figure 2**. We found that naïve Th cells were significantly increased in both the prepubertal and pubertal cohort (**Figure 3**; **Supplemental Table 2**). PD1 expression on naïve Th cells continued to have no significance on the pediatric subpopulation analysis. Interestingly, the significant decrease in CD31^+^ (RTE) naïve T cells in the overall pediatric group was due primarily to a decrease in the prepubertal group (**Figure 3**; **Supplemental Table 2**), with an effect ratio of 0.5 in the prepubertal children (**Figure 3**), increasing to an effect ratio of 1.2 in the pubertal group, which approaches that of the adults (effect ratio of 2.1). While we identified no difference in T_reg_ populations in the overall pediatric cohort, our sub analysis identified that PD1^-^ memory T_reg_ cells were increased in the prepubertal group (**Figure 3**; **Supplemental Table 2**), and PD1^+^ memory T_reg_ cells were significantly increased in the pubertal group. A second T_reg_ population was altered in the prepubertal group, with a decrease in RTE (CD31^+^) naïve T_reg_ cells, whereas these cells were not significantly different in the pubertal group.

We evaluated the impact of puberty at the time of HSCT on B cell populations at day 100 in the ABLE cohort. We selected the six B cell populations that were significantly different between the adult and pediatric cohorts (excluding mature naïve B cells, as they were decreased in both adults and children). We found that the T2 and T3 transitional B cell populations were only decreased in prepubertal children, but were not significantly different in the pubertal population. In prepubertal populations, the effect ratios of 0.44 and 0.44 for the T2 and T3 transitional B cell populations, respectively, were significant; however, neither were significant for the pubertal population. (**Figure 3**; **Supplemental Table 2**). Evaluation of the unswitched memory/marginal zone-like B cells demonstrated that these B cells were only significantly increased in prepubertal children (effect ratio of 3.7) and had a non-significantly lower effect ratio (1.4) in the pubertal population. By contrast, we saw a significantly decreased effect ratio in of CD21^low^ B cells in prepubertal with a non-significant decrease in this population pubertal children to 0.53. Switched memory B cells continued to be unchanged in cGvHD in the pediatric cohort.

NK_reg_ cells were decreased with cGvHD (**Figure 3**) in both the prepubertal group with an effect ratio of 0.67 (p = 0.006; ROC AUC = 0.68) and for pubertal children with an effect ratio of 0.68 (p = 0.05; ROC AUC = 0.69). The impact of puberty pre HCT on cytokines expression in plasma was also evaluated (**Figure 3**; **Supplemental Table 2**). ST2 was significantly elevated in both prepubertal children with an effect ratio of 1.5 (p = 0.04; ROC AUC = 0.68) and pubertal children with an effect ratio of 1.5 (p = 0.04 ROC AUC = 0.68). By contrast, Aminopeptidase N or sCD13 was only significantly increased in prepubertal children with an effect ratio of 1.6 (p = 0.004; ROC AUC 0.67) and non-significantly increased in pubertal children with an effect ratio of 1.3 (p = 0.11; ROC AUC = 0.69). Similar to sCD13, ICAM1 was only significantly elevated in prepubertal children with cGvHD with an effect ratio of 1.4 (p = 0.01; ROC AUC = 0.63). Neither CXCL10 or CXCL9 were increased in either group.

## Discussion

Although there appear to be consistent cGvHD immune profile patterns in NK_reg_ cells, naïve Th cells, and ST2, regardless of age (**Table 3**), the comparisons we have performed support that significant differences exist in a number of T and B cell subpopulations and cytokine profile patterns between pediatric and adult HSCT patients with cGvHD. For cellular populations that were different between the pediatric and adult cohorts in our studies, pubertal patients often had immune profile values in-between adults and prepubertal children (**Table 3**), suggesting a transition in cGvHD mechanisms associated with the onset of puberty.

**Table 3:** Summary of Recipient Age on cGvHD markers.

	Pre pubertal	Pubertal^1^	Adult
**Naïve T cells**			
Naïve Th cells	Increased	Increased(NS)	Increased
RTE Naïve Th cells	Decreased	NS	Increased^2^
PD1- or PD1+ Naïve Th cells	NS	NS	Increased
**Memory T cells**			
PD1^+^ memory Th cells	NS	NS	Decreased
PD1- Naïve Th cells	NS	NS	Increased
**Newly formed B cells**			
CD21^lo^ B cells	Decreased	NS	Increased
T2 transitional	Decreased	NS	NS
T3 transitional	Decreased	NS	NS
**Peripheral B cells**			
Mature Naïve	Decreased	NS	Decreased
Unswitched memory/Marginal-zone like	Increased	Increased(NS)^3^	Decreased
Classical switched memory	NS	Increased(NS)	Increased
**Regulatory populations**			
**Regulatory T cells**			
PD1^-^ memory T_reg_ cells	Increased	Decreased(NS)	NS
PD1+ memory T_reg_ cells	NS.	Increased	NS
RTE memory T_reg_	Decreased	NS	NS
PD1- Naïve Treg	NS	NS	Increased
RTE Naïve Treg	NS	Increased(NS)	Increased
**Regulatory NK cells**	Decreased	Decreased	Decreased
**T1 - Transitional population consistent with Breg cells**	NS	NS	Decreased
**Cytokines and Chemokines**			
ST2	Increased	Increased	Increased
CXCL10	NS	NS	Increased
CXCL9	NS	NS	Variable
Aminopeptidase N (sCD13)	Increased	Increased (NS)	Variable
ICAM-1	Increased	NS	NS

^1^Prepubertal was defined as a girl aged <10.9 years or boy <12.4 years at time of HSCT and pubertal was defined as a girl ≥10.9 years or boy ≥12.4 years at time of HSCT. ^2^CD31 expression was not important in adults with both CD31^+^ and CD31^-^ Th cell populations elevated. ^3^the effect ratio met criteria of either ≤0.75 or ≥1.3 but was not statistically significant due to smaller number of patients.

### Age Related T Cell Differences

T cell similarities and differences identified appear to involve primarily Naïve Th cells (**Table 3**). Thymic function is highest before puberty, with intermediate function at the onset of puberty and further decline towards adulthood (10). Both children and adults in our cohorts had significant expansions of naïve Th cell associated with cGvHD, although the pattern of naïve Th cell expansion was different. In adults, we observed that PD1 expression in naïve Th cells may play a role in the development of cGvHD, with an expansion of PD1^-^ naïve Th cells (**Figure 2**). By contrast, PD1 expression on naïve Th cells was not important in the cGvHD pediatric cohort. One other difference in the T cell pattern we observed between pediatric and adult cohorts was that in adults (but not children) PD1^-^ memory Th cells were increased, whereas PD1^+^ memory Th cells were decreased. This would be consistent with the dependence on PD1 as a regulator of peripheral tolerance, the primary mechanism of T cell tolerance in adult and post pubertal recipients (22). PD1 independent and thymic-dependent mechanisms may therefore play a greater role in the prepubertal population in regulating cGvHD development.

### Age Related B Cell Differences

Our group was the first to identify the critical role of B cells in the development of cGvHD, in mice (23), followed by identification of significant B cell abnormalities in children (24). Interestingly, in the current analysis, the B cell compartment is where we identified no common B cell profiles and some of the greatest differences between the pediatric and adult cohort (**Table 3**). The primary impact of cGvHD, both in adults and children, appears to be in what has been recently designated as newly formed B cells (NF-B), including all transitional and immature B cells (25). In the pediatric cohort, we saw a broad depression of the NF-B cell populations associated with cGvHD including the T1-CD21^lo^, T2, T3, and mature naive B cell populations, with these differences primarily seen in the prepubertal subgroup. The only population that was increased in cGvHD in the pediatric group was the unswitched memory/marginal zone-like B cell population. In contrast to the pediatric group, adults had a significant increase in the transitional T1- CD21^lo^ B cell population (associated with autoimmunity) and a significant decrease in transitional T1- CD24^hi^CD38^hi^ B cell populations (associated with a B_reg_ population). This suggests that B_regs_ may play a much greater role in controlling cGvHD in adults compared to children.

The different B cell patterns seen in NF-B cells between children and adults suggests there are significant differences in the role that B cell regulation may play in pediatric versus adult cGvHD. There is evidence that patients with autoimmune disease suffer from defects in early B‐cell tolerance checkpoints in the T1 transitional NF-B cell populations, resulting in selection of autoreactive NF-B cells (26, 27) that potentially present self‐antigen to T cells, similar to early murine models of cGvHD (23). In the area of autoimmunity, increased circulating NF–B cells are found in SLE, type 1 diabetes, and juvenile dermatomyositis (28, 29) possibly as an important source of pathogenic autoantibodies. It is suggested that autoreactive NF-B cells contains clones that may develop into CD27^−^ CD21^−/lo^ B cells after the acquisition of somatic hypermutations that improve affinity for self‐antigens (27). In adults, we observed an inverse relationship between increased CD21^lo^ B cells and decreased T1 B_regs,_ suggesting a possible inhibitory impact of B_reg_ cells on CD21^lo^ B cells at the early B cell checkpoint.

In children, we saw a very different pattern that also appeared to be a result of an early B cell checkpoint abnormalities that resulted in a broad suppression of a number of NF-B cell populations including CD21^lo^ B cells. These two different patterns of B cell abnormalities resulted in increased classic switched memory and decreased unswitched B cell in adults and increased unswitched memory B cell in children. NF–B cell activation appears to be driven by TLR7 and TLR9 activation by recognition of RNA and DNA motifs (30–32). Our previous observation that a TLR-9 responsive B cell population was associated with the onset of cGvHD in children (24) suggests an aberrant NF-B population in children. In children, there is one other possible mechanisms by which B cell may impact on development of post HSCT tolerance whereby intrathymic B cells may support development of T_regs_ through cognate help and can shape the T_reg_ repertoire (33).

### Regulatory Cells in cGvHD

Of all of the regulatory populations, NK_reg_ cells (19) appear to have a consistent age-independent role in suppression of cGvHD. CD56^bright^ NK cells represent 10% of peripheral NK cells and are similar to decidual NK cells, with regulatory function, that inhibit placental rejection (34). The NK_reg_ populations is characterized by expression of granzyme K rather than expression of either perforin or granzyme B (35) and many times are considered as non-cytolytic. With large patient populations, we have identified in both children and adults, a non-cytolytic CD56^bright^ NK population (NK_reg_) closely correlating with a lack (or inhibition) of cGvHD. In adults, we have seen increased CD56^bright^ CD335^+^ CXCR3^+^ NK_reg_ cells associated with decreased cGvHD (4); increased CD56^bright^ NK_reg_ cells in adult donor product that correlated with suppression of cGvHD (3); and increased NK_reg_ cell numbers induced by ATG-treatment day at 100 post HSCT in 38 adults on the CBMTG 0801 trial where ATG significantly decreased cGvHD (12). Similarly, in the pediatric ABLE studies, we found increased noncytolytic CD56^bright^ NK_reg_ cells in pediatric recipients at 3 months post HSCT in those who did not later develop cGvHD (8).

It has been postulated that T_reg_ cells play a role in cGvHD, but there are many conflicting studies regarding their role (36–38). Part of differing findings may be a result of age-related differences in the role of T_reg_ cell subpopulations in cGvHD (**Table 3**). We found that differences in memory T_reg_ cells were more predominant in prepubertal and pubertal groups whereas naïve T_reg_ cells appeared to be more important in adults (**Table 3**). In the prepubertal group we observed both an increase in PD1^-^ memory T_reg_ cells numbers and a decrease in CD31^+^, RTE memory T_reg_ cells (**Table 3**). Interestingly, a previous analysis of our group of the pediatric cohort at day 100 in those patient that had already developed cGvHD found an increase in PD1^-^ memory T_reg_ cells and a concomitant decrease in PD1^+^ memory T_reg_ cells (8). By contrast adults had no observable differences in memory T_reg_ cells but did have an increase in naïve T_reg_ cells either expressing CD31 (RTE) or lacking PD1 expression (**Figure 2**, **Table 3**).

The role of PD1 in regulation of memory and naive T_reg_ cells appears to be inadequate where PD1 blockade increases the proliferation of highly suppressive PD1^+^ memory T_reg_ cells and inhibition of antitumor immunity (25). The absence of PD1 along with partial FoxP3 insufficiency, however, can result in T_reg_ cells with proinflammatory properties and expansion of effector/memory T cells that contributed to the autoimmunity (39). Others have established that PD1 is critical in modulating T_reg_ homeostasis during low does IL-2 therapy for cGvHD (41). They observed that PD1^-^ memory T_regs_ showed rapid Stat5 phosphorylation and proliferation with IL-2 initiation followed by higher Fas and lower Bcl-2 expression decreased the effectiveness of IL-2 on memory T_regs_ (40). The importance of PD1 expression on either memory or naïve T_reg_ cells is further supported by a study that demonstrated that PD1 upregulated on T_reg_ cells and its interaction with PD1 ligand on effector T cells resulted in potent T cell suppression (41).

The other regulatory population that may be more prominent in the adult population is the T1- CD24^hi^CD38^hi^ B cell population, many times associated with a B_reg_ function. While we acknowledge that the only definitive way to evaluate B_reg_ cells is by a functional assay and thus cannot be sure this T1 B cell population is in fact a B_reg_ cell population, we could identify only a decrease in the B_reg_ phenotype in adults and not in children.

### Age Related Cytokine Differences

Soluble ST2 was the only cytokine consistently increased in all age groups. Elevation in ST2 associated with cGvHD has been described in multiple adult studies and in our previous ABLE pediatric study (4, 5, 8, 22). The ST2-related chemokines, CXCL9 and CXCL10 were not elevated in the younger population suggesting a greater role in adults. By contrast, two cytokines may play a greater role in the pediatric population, aminopeptidase N (sCD13) and ICAM-1 (**Figure 3**), neither which is associated with ST2 functionally. We had initially found elevation of sCD13 in a pediatric study, COG ASCT0031, through proteomic discovery and validation (21). Subsequently, we and others have seen an elevation of sCD13 in adults. Our current analysis of the prepubertal versus the pubertal group showed that the association was strongest in prepubertal children. The source of sCD13 in the HSCT cGvHD environment is still not known. One group did find an apparently distinct CD13^+^CD33^+^ population of leukemic cells contributing to a proinflammatory microenvironment that was detrimental to long-term normal hematopoiesis (42) suggesting an inflammatory role of sCD13 in the hematopoietic microenvironment. It is possible that the increased sCD13 may impact early B cell lymphopoiesis in cGvHD. Support for this hypothesis is provided by data demonstrating the impact of Bestatin, a sCD13 inhibitor, on B cell lymphopoiesis. In mice, treatment with Bestatin increased the total number of thymocytes, splenocytes, and lymphocytes of mesenteric lymph nodes. Inhibition of sCD13 by bestatin decreased peripheral Th and Tc cells and augmented B cells in the peripheral lymphatic sites (43). In humans, Bestatin had a similar effect, augmenting immune reconstitution following HSCT with significant increases in NK cells and B cells (44). Thus, it is possible that the suppression of NF-B lymphopoiesis and thymopoiesis seen in children with cGvHD may be impacted by elevations of sCD13.

### Impact of Sex Hormones on Immune Function in HSCT and cGvHD

It is well established that the onset of puberty appears to initiate the involution of the thymus and a decrease in thymic function (10). The thymus involutes after estradiol treatment or during pregnancy, in both mice and humans (45, 46). Since testosterone partially is converted to estradiol, testosterone treatment probably also has an identical impact. Moreover, decreasing recipient thymic function is hypothesized to be one of the major reasons for the increase in cGvHD seen in adult compared to children. Some have proposed that using steroid ablation will result in thymic regeneration in adults and potentially both help in both immune reconstitution after HSCT, but also may impact on the development of cGvHD (47). Our studies are limited in that we evaluated the pubertal status at the time of HSCT and could not evaluate the impact of hormonal levels on immune reconstitution after HSCT. We know that all myeloablative preparative regimens, whether including total body irradiation (TBI). However, steroid-secreting cells (Leydig cells in boys and granulosa cells in girls) are more resistant to TBI and high-dose cytotoxic drugs. Whereas germ cell producing Sertoli cells, in boys, and oocytes, in girls, are much more sensitive (48). It is also possible that alteration in sex hormone levels post HSCT may impact on immune reconstitution and possible the onset of cGvHD. We attempted to accommodate for some of these variables in our analysis of the prepubertal and pubertal sub analysis by considering a number of clinical covariates including both TBI and time of cGvHD onset as covariates and none of these factors had a major impact on the final results.

### Proposed Model for Age Related Differences in cGvHD Immune Profiles

**Figure 4:**
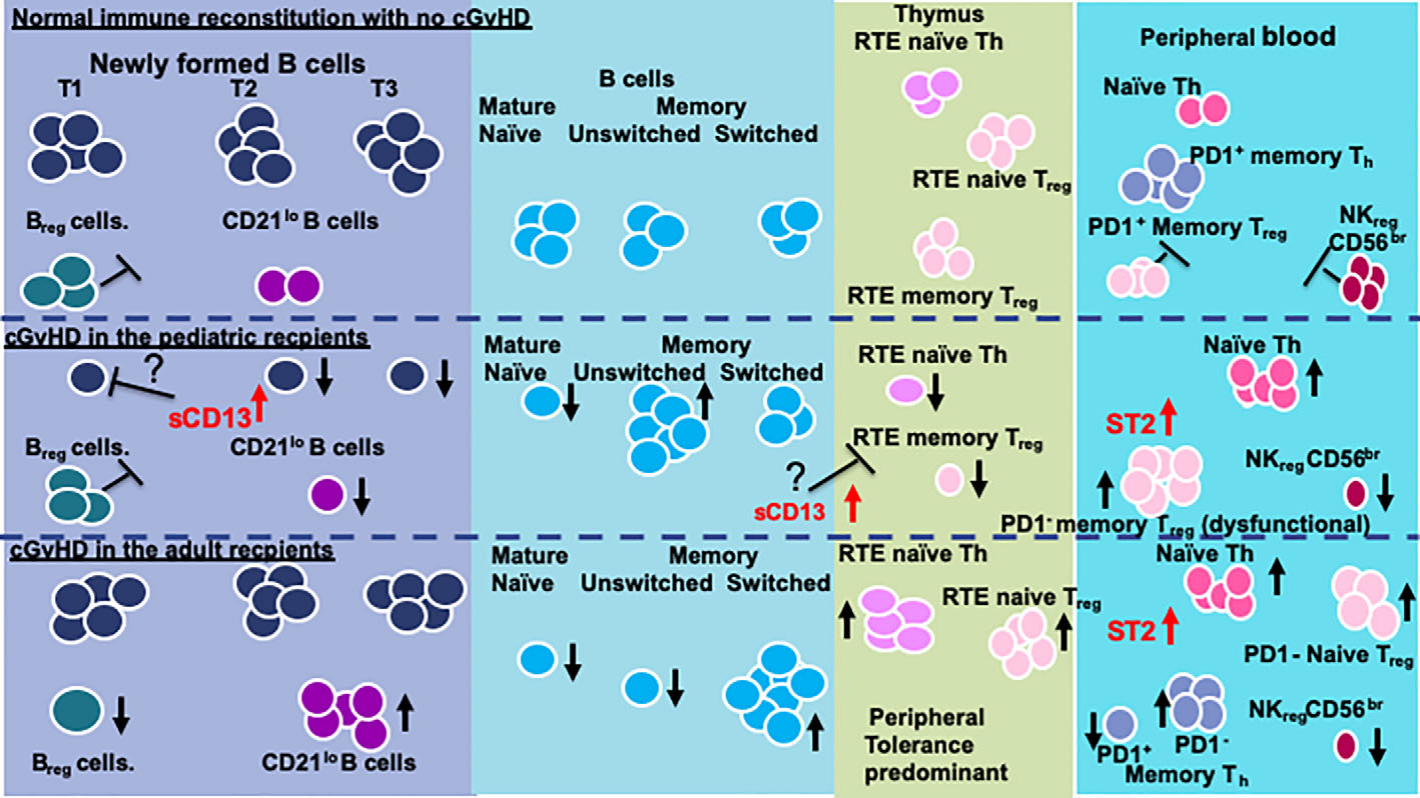
Model of the differences in between children and adults.

While many questions remain, we conclude that recipient age at the time of HSCT impacts on the immune profile of cGvHD cell populations and cytokines and needs to be taken into consideration when evaluating the biomarkers and immunology of cGvHD. We summarize the age related differences we have been able to identify and have attempted to develop a working model for the recipient impose differences in cGvHD development (**Figure 4**). In general, there appears to be some major differences in the immune profiles of pediatric versus adult cGvHD. In the T cell compartment, all age groups appear to have an increase in naïve Th cells. Interestingly, the increase in adults seem to be primarily due to an increase in an RTE PD1^-^ naïve Th cell population. We saw more striking differences in B cells (**Figure 4**). Children had a broad suppression of NF-B cells where as many of the difference in adults appeared to be at the T1 transitional stage with an increase in CD21^lo^ B cells that probably are due to an aberrant early B cell check point inhibition that may have prevented the development of this population. We hypothesize that there may be age related differences in how B cell abnormalities develop in NF-B cells at the early checkpoint associated with T1-trasnional B cells. In adults, there appears to be a major divergence to increased CD21^low^ B cells whereas children develop a broad suppression of almost all NF-B cell lymphopoiesis.

Three regulatory populations appear to be important in preventing the development of cGvHD. All age groups appeared to depend on NK_reg_ cell suppression of cGvHD, but only adults appeared to have an association with cGvHD suppression by a T1 transitional B cell population that phenotypically is similar to B_reg_ cells (49). Children seem to develop abnormalities primarily in memory T_reg_ cells whereas adults had abnormalities in naïve T_reg_ cells. These findings suggest that the dynamics of the regulatory losses may be different before and after puberty. While ST2 is consistently elevated regardless of age, aminopeptidase N (sCD13), which is not known to be affected by ST2, was more prominently elevated in the prepubertal group and we hypothesize may be impacting on both dysfunctional B cell lymphopoiesis and thymopoiesis.

One factor that may have influenced the comparison of adult to pediatric cGvHD biology are the differences in HSCT approaches between the two age cohorts including the greater use of myeloablative regimens, higher number of non-malignant indications, and low use of PBPC as a donor source in the pediatric population compared to adults. In our multivariate analyses, we corrected for these co-variates as much as possible and still observed significant differences leading to our conclusion that there are recipient age-related differences. Moreover, our comparison of the prepubertal to the pubertal subgroups essentially compared groups receiving he identical transplant approaches as they were limited to pediatric HSCT centers.

In summary, these data support that the impact of pre HSCT age and pubertal development may both explain why children have a lower cGvHD, possible different organ distribution, and most importantly have distinct biological differences in some of the pathways that result in the development of cGvHD. One thing to emphasize in that we many times found cell population patterns in the pubertal group somewhere between that in prepubertal children and adults suggesting that the onset of puberty begins to affect cGvHD patterns somewhere between children and adults. Moreover, the impact of post HSCT sex hormone production on the development if immune tolerance and cGvHD is also probably impacted by the pre HSCT pubertal status. We have tried to develop a model that only partially explains the observed differences and more information is required.

## Data Availability Statement

The raw data supporting the conclusions of this article will be made available by the authors, without undue reservation.

## Ethics Statement

The studies involving human participants were reviewed and approved by University of British Columbia. Written informed consent to participate in this study was provided by the participant or the participant’s legal guardian/next of kin.

## Author Contributions

GC: clinical lead of the pediatric ABLE studies with study performance, interpretation of data, and writing. AL: interpretation of T cell analysis and writing of the manuscript. SD: interpretation of T cell analysis and writing of the manuscript. AK: performance of all analyses, interpretation of results, and writing of the manuscript. SA: performance of all analyses, interpretation of results, and writing of the manuscript. JR: performance of B cell studies, interpretation of the results, and writing. BN: performance of all pediatric cohort statistical analyses and writing. SM: oversight of all statistical analyses and writing. RB: oversight of all T cell analyses, interpretation, and writing. KS: overall analysis, study design, performance, interpretation, and writing. All authors contributed to the article and approved the submitted version. J-PC is a pediatric endocrinologist. His contributions to the present paper include: Substantial contributions to the interpretation of data for the work (puberty); Revising the work critically for important intellectual content in the endocrine area (puberty, hormones); Final approval of the version to be published; Agreement to be accountable for all aspects of the work in ensuring that questions related to the accuracy or integrity of any part of the work are appropriately investigated and resolved.

## Funding

Canadian Institute of Health Research Canadian Cancer Society Leukemia Lymphoma Society of Canada Garron Foundation. This work was funded by Canadian Institutes of Health Research (CIHR) operating, Team, and Foundation grants, Leukemia Lymphoma Society of Canada, Garron Foundation, Canadian Cancer Society.

## Conflict of Interest

The authors declare that the research was conducted in the absence of any commercial or financial relationships that could be construed as a potential conflict of interest.
