# Supplementary material for: “Age Related Differences in the Biology of Chronic Graft-Versus-Host Disease After Hematopoietic Stem Cell Transplantation”
Source: Front Immunol. 2020 Oct 16;11:571884. doi: 10.3389/fimmu.2020.571884 (PMC7641628; doi:10.3389/fimmu.2020.571884)
Supplement: Supplementary file 1 [file DataSheet_1.pdf]

Supplemental Table 1. Direct Comparison Analysis of Adult versus pediatric Cohorts for T cell populations at Measured at day 100 after HSCT

Cell population		Pediatric – (N = 241)[2]	Adult – (N = 97)[3]
**Naive T cell populations**			
Naïve Th cells	$CD31^{-}CD45RA^{+}CD4^{+}$	**Increased**<br>1.6 (0.02)<br>0.68	**Increased**<br>2.0 (0.003) [1]<br>0.68
RTE Naïve Th cells	$CD31^{+}CD45RA^{+}CD4^{+}$	**Decreased (NS)**<br>0.67 (0.05)<br>0.59	**Increased**<br>2.0 (p = 0.009) [1]<br>0.66
$PD1^{-}$ Naïve Th cells	$PD1^{-}CD4^{+}CD45RA^{+}$	**NS**<br>In cGvHD	**Increased**<br>1.9 (0.002)<br>0.69
**Memory T cell populations**			
$PD1^{-}$ memory Th cells	$PD1^{-}CD4^{+}CD45RA^{-}$	**NS**<br>In cGvHD	**Increased**<br>1.3 (0.003)<br>0.68
$PD1^{+}$ Memory Th cells	$PD1^{+}CD4^{+}CD45RA^{-}$	**NS**<br>In cGvHD	**Decreased**<br>0.70 (0.0007)<br>0.70
**Treg cells**			
All Memory T_{reg} populations	$CD45RA^{-}CD4^{+}CD25^{+}CD127^{-}$	**NS**<br>In cGvHD	**NS**<br>in cGvHD
Naïve T_{reg} populations	$PD1^{-}CD45RA^{+}CD4^{+}CD25^{+}CD127^{-}$	**NS**<br>In cGvHD	2.4 (0.0006)<br>0.70
	$CD31^{+}CD45RA^{+}CD4^{+}CD25^{+}CD127^{-}$	**NS**<br>In cGvHD	1.6 (0.02)<br>0.63

[1]These cohorts had a significantly increased effect ratio and ROC AUC value but the p value by Wilcoxon Rank Sum did not meet significance; [2]Data from reference [7, 8, 22]; [3]Data from reference [4,19]

Supplemental Table 2. Subpopulation Comparison Analysis Based on the Estimated pubertal state at the time of HSCT on cGvHD markers associated with cGvHD at day 100 in the ABLE pediatric Cohort[2]			
		**Estimated as Prepubertal <10.9 years boys <12.4 years girls**	**Estimated as Pubertal ≥10.9 years boys ≥12.4 years girls**
**B cell populations**			
T1 –Transitional consistent with Breg cells	$CD10^{hi}CD38^{hi}CD19^{+}$	**NS**	**NS**
CD21 low B cells	$CD21^{lo}$/CD19cells[2]	**Decreased** 0.38 (0.02) 0.80	**NS**
T2 transitional	$CD38^{int}CD10^{int}$ B cells	**Decreased** 0.44 (0.002) 0.74	**NS**
T3 transitional	$CD38^{dim}CD10^{lo}$B cells	**Decreased** 0.44 (0.0001) 0.78	**NS**
Mature Naïve B cells	IgD+CD27-	**Decreased** 0.65 (0.0004) 0.79	**NS**
Unswitched memory/ Marginal-zone like	$CD27^{+}$ IgD^{+} of $CD19^{+}$	**Increased** 3.7 (0.01) 0.62	**NS**
Classical switched memory	$CD27^{+}IgD^{-}$ of $CD19^{+}$	**NS**	**NS**
**Regulatory NK cells (noncytolytic)**			
NKreg cells	CD56hiPlo	**Decreased** 0.67 (0.006) 0.68	**Decreased** 0.68 (0.05) 0.69
**Plasma markers**			
Aminopeptidase N (sCD13)		**Increased** 1.6 (0.004) 0.67	**Increased (NS**) 1.3 (0.11) 0.58
ST2		**Increased** 1.5 (0.04) 0.68	**Increased** 1.5 (0.04) 0.68
CXCL10 or CXCL9		**NS**	**NS**
ICAM1		**Increased** 1.4 (0.01) 0.63	**NS**
**Naïve T cells**			
Naïve Th cells	$CD4^{+}CD31^{-}CD45RA^{+}$	**Increased** 1.5(0.04) 0.61	**NS**
RTE Naïve Th cells	$CD4^{+}CD31^{+}CD45RA^{+}$	**Decreased** 0.5 (0.03) 0.65	**NS**
PD1⁻ Naïve Th cells	$CD4^{+}CD45RA^{-}PD1^{-}$	**NS**	**NS**
**Memory T cells**			
PD1+ memory Th cells	$CD4^{+}CD45RA^{-}PD1^{+}$	**NS**	**NS**
PD1- memory Th cells	$CD4^{+}CD45RA^{-}PD1^{-}$	**NS**	NS
**Treg cells**			
PD1- Memory Treg	$PD1^{-}CD45RA^{-}$ $CD4^{+}CD25^{+}CD127^{-}$	**Increased** 1.3 (0.02) 0.68	**Decreased NS**[3]
PD1+ Memory Treg	$PD1^{+}CD45RA^{-}$ $CD4^{+}CD25^{+}CD127^{-}$	**NS**	**Increased** 1.6 (0.009) 0.71
RTE Naïve Treg	$CD31^{+}CD45RA^{+}CD4^{+}$ $CD25^{+}$ $CD127^{-}$	**Decreased** 0.44 (0.03) 0.65	**NS**

[1]The Pubertal status was based on the estimated Tanner staging of the average genital stage 3 for boys at 12.4 years and breast stage 2 at 10.9 years in girls. These Tanner stagers were selected as the onset of accelerated height velocity in the two sexes and the averages selected based on North American standards **[16]**. [2]Adjusted for the impact of TBI and time of onset of cGvHD post HSCT[3]Increased NS or decreased NS means the value was not significant but trending toward the adult ratio

Supplemental Table 3. Summary of antibodies used in flow cytometric analysis for both the previously published manuscripts and for the current prospective analyses presented in the manuscript.				
	**Dye**	**AB**	**Cat No. / REF No.**	**Manufacturer**
1	AlexaFluor 488	CD4	344618	Biolegend
2	APC-Cy7	CD3	300426	Biolegend
3	PE	CD197 (CCR7)	353204	Biolegend
4	PerCPCy5.5	CD127	351322	Biolegend
5	PE	CD337	325208	Biolegend
6	BV421	CD335	331914	Biolegend
7	BV510	IgD	348220	Biolegend
8	FITC	CD21	354910	Biolegend
9	PE	CD25	356104	Biolegend
10	APC-eFluor 780	CD14	47-0149-42	eBioscience
11	BV421	CD13	582598	BD
12	BV785	CD19	302240	Biolegend
13	Pacific Blue	CD19	302232	Biolegend
14	AlexaFluor 647	CD45	304018	Biolegend
15	AlexaFluor 647	CD69	310918	Biolegend
16	PE	CD66b	305105	Biolegend
17	AlexaFluor 488	CD56	304611	Biolegend
18	PE	CD56	304606	Biolegend
19	FITC	CD45RA	304106	Biolegend
20	PB	CD45RA	304123	BioLegend
21	BV785	CD27	302832	Biolegend
22	BV421	CD31	303124	Biolegend
23	PE	CD38	303506	Biolegend
24	APC	CD10	312210	Biolegend
25	PerCP	CD5	300618	Biolegend
26	PerCP	CD3	300428	Biolegend
27	Pacific Blue	CD8a	301033	Biolegend
28	BV510	CD8a	301048	Biolegend
29	BV785	CD4	317442	Biolegend
30	AlexaFluor488	Perforin	308108	Biolegend
31	AlexaFluor647	GranzymeB	515406	BD
32	AlexaFluor 647	PD-1(CD279)	329910	Biolegend
